# Applying supervised machine learning algorithms and ensemble models to enhance credit card fraud detection

**DOI:** 10.3389/frai.2026.1813728

**Published:** 2026-05-25

**Authors:** Abrar Al-Bulushi, Abdul K. Shaikh, Naresh Adhikari

**Affiliations:** 1 Sohar International Bank, Muscat, Oman; 2 Department of Information Systems, Sultan Qaboos University, Muscat, Oman; 3 Department of Computer Science, Slippery Rock University, Slippery Rock, PA, United States

**Keywords:** artificial intelligence, bagging, ensemble learning, fraud detection, imbalanced data handling, supervised machine learning

## Abstract

Credit card fraud has become a significant threat to individuals, financial institutions, businesses, and governments, causing substantial annual economic losses through increasingly sophisticated fraudulent activities. This research aims to enhance credit card fraud detection by leveraging machine learning algorithms and ensemble learning techniques to improve detection accuracy and model robustness. Various supervised machine learning algorithms were implemented, including Decision Tree (DT), Logistic Regression (LR), Naïve Bayes (NB), Random Forest (RF), Artificial Neural Network (ANN), and Extreme Gradient Boosting (XGBoost). To address the issue of highly imbalanced datasets, multiple resampling techniques were applied, including Random Under-Sampling (RUS), Random Over-Sampling (ROS), and Synthetic Minority Over-Sampling Technique (SMOTE). Feature selection and data preprocessing techniques were also incorporated to improve model performance. The models were evaluated using several performance metrics, including accuracy, precision, recall, F1-score, and Area Under the Curve (AUC). Additional optimization was performed through threshold variation on the best-performing models, namely Random Forest and XGBoost. Furthermore, ensemble learning techniques, including bagging, boosting, and stacking, were employed to enhance fraud prediction capability and improve model generalization. The results demonstrated that the ensemble learning models significantly improved fraud detection performance compared to standalone models. Among the evaluated approaches, the bagging ensemble technique achieved the best overall performance, obtaining 0.99 accuracy, 0.90 recall, and 0.77 precision. The ensemble model combined Decision Tree, Random Forest, and Artificial Neural Network base learners using different resampling techniques, which increased model diversity and robustness. The proposed approach also maintained strong performance when evaluated on unseen datasets, demonstrating its generalization capability. The findings indicate that integrating ensemble learning, resampling strategies, and multiple machine learning models can substantially improve credit card fraud detection systems. The proposed framework provides a robust and scalable approach for detecting fraudulent transactions while maintaining high predictive performance in real-world imbalanced financial datasets.

## 1 Introduction

Credit cards are widely used worldwide for various purposes, such as shopping, traveling, and online purchases. They offer several benefits, including convenience, security, and reward programs. The prevalence of e-commerce and mobile devices has led to an increase in the use of credit cards, allowing consumers to make online transactions more efficiently (Kiselichki et al., 2022). However, as credit cards have become the most popular mode of payment, the number of associated fraud cases has also risen (Bhanusri et al., 2020). According to the Nilsson report, a well-known source of card and mobile payment news, global losses from payment card fraud were $27.85 billion in 2018 and are expected to increase to $35.67 billion by 2023 (Nilson, 2019). Moreover, based on the United States Federal Trade Commission, a government agency for consumer protection, in 2023, Americans fell victim to fraud, resulting in a loss exceeding $10 billion, indicating a 14% surge in reported losses from the preceding year (Theng and Bhoyar, 2024).

Credit card fraud refers to the unauthorized use of a credit card or its information for illegal transactions (Sailusha et al., 2020). Fraud can occur in various forms, including physical card theft, card skimming, and phishing attacks (Jain et al., 2019). With advancements in digital banking, fraud techniques continue to evolve, making detection increasingly challenging. The financial consequences of such frauds are substantial and expected to grow, underscoring the need for advanced machine-learning-based detection systems (TrivediVarmedja et al., 2019).

Machine learning, defined as the development of statistical algorithms that learn patterns from data and generalize to unseen cases without explicit programming (Koza et al., 1996), plays a critical role in fraud detection. Depending on the dataset characteristics, either supervised or unsupervised learning methods can be utilized. Common supervised models include Decision Trees (DT), Naïve Bayes (NB), Least Squares Regression (LSR), Logistic Regression (LR), and Support Vector Machines (SVM) (Dornadula and Geetha, 2019). These models are trained on historical transaction data, including known fraudulent events, to classify new transactions as legitimate or suspicious. Transactions flagged as suspicious are subjected to further verification (Khatri et al., 2020).

Detecting credit card fraud remains difficult due to several challenges. The most significant is the highly imbalanced nature of transaction datasets, where fraudulent cases constitute only a small fraction of the total. This imbalance biases machine learning models toward the majority class, reducing detection accuracy (Kalid et al., 2020). Furthermore, fraudsters continually adapt their techniques, requiring ongoing updates and monitoring of detection algorithms (Dornadula and Geetha, 2019). The increasing volume and complexity of real-time transaction data also make timely fraud detection difficult, often causing delays in identifying malicious activities (Sailusha et al., 2020).

Despite the extensive application of machine learning and ensemble techniques in credit card fraud detection, several research gaps remain. Existing studies often focus on single datasets, lack robustness testing on multiple unseen datasets, or do not explore the combined impact of feature engineering, resampling strategies, and ensemble diversity on model generalization. Additionally, limited attention has been given to behavioral feature engineering and threshold optimization in improving fraud detection performance.

The main contributions of this study are summarized as follows:

1. Development of a comprehensive fraud detection framework using multiple supervised machine learning models combined with ensemble techniques.
2. Integration of novel behavioral features (e.g., transaction frequency and time-based anomaly indicators) to enhance model performance.
3. Systematic evaluation of multiple resampling techniques (RUS, ROS, and SMOTE) to address class imbalance.
4. Implementation of ensemble models (bagging, boosting, and stacking) to improve robustness and predictive accuracy.
5. Validation of model generalization using multiple unseen datasets from diverse sources.
6. Application of threshold tuning to optimize recall–precision trade-offs in fraud detection.

While prior studies have explored machine learning and ensemble techniques for fraud detection, most focus on isolated improvements such as algorithm comparison or single-dataset evaluation. In contrast, this study proposes an integrated framework that combines multi-dataset validation, behavioral feature engineering, resampling strategies, and ensemble diversity within a unified pipeline. This holistic approach enables a more comprehensive evaluation of model robustness and generalization capability, particularly across unseen datasets with varying characteristics

The remainder of this paper is organized as follows: Section 2 reviews previous work in the field of credit card fraud detection. Section 3 presents the methodology and conceptual framework. Sections 4, 5 discuss the experiments and their results. Section 6 offers recommendations and outlines potential future work. Finally, Section 7 provides concluding remarks.

## 2 Literature review

### 2.1 Machine learning algorithms' application on credit card fraud detection

Recent studies have increasingly focused on enhancing fraud detection performance through both model-centric and data-centric approaches. For example, (Aslam and Hussain 2024) demonstrated that ensemble learning techniques consistently outperform standalone models in terms of accuracy and robustness, highlighting the importance of combining multiple learners. Similarly, (Hussain et al. 2024) emphasized the critical role of resampling strategies, showing that both over-sampling and under-sampling techniques significantly improve recall in highly imbalanced datasets.

More recent advancements have shifted toward addressing deeper data-related challenges. (Wu et al. 2025) introduced the concept of informative missingness in semi-supervised learning, illustrating how missing data patterns can carry meaningful information and improve model performance when properly leveraged. In parallel, (Zhang et al. 2025) proposed a margin-enhanced data augmentation method that generates more informative synthetic samples, improving class separability in imbalanced credit prediction tasks.

(Afriyie et al. 2023) examined the effectiveness of supervised learning techniques in identifying and predicting fraudulent credit card transactions. Their study compared three classification models: Decision Tree (DT), Logistic Regression (LR), and Random Forest (RF). The results demonstrated that the RF model achieved superior performance compared to LR and DT, showing higher accuracy, sensitivity, and specificity in detecting fraudulent activities.

(Serongwa 2023) conducted a study on credit card fraud detection systems using eXtreme Gradient Boosting (XGBoost) and Isolation Forest (iForest). The study emphasized the significance of model evaluation metrics and highlighted the importance of feature engineering in enhancing the performance of fraud detection systems. The study found that the XGBoost model outperformed the iForest model in terms of predictive power and scalability. The XGBoost model demonstrated superior performance in detecting credit card fraud compared to the iForest model.

In a study conducted by (Jain et al. 2020), the authors explored the implementation of machine-learning algorithms in credit card fraud detection. They compared the performance of three algorithms, DT, RF, and XGBoost, on a dataset containing information from 284,808 credit cards. The results showed that XGBoost had the highest accuracy at 99.962%, while DT had the lowest accuracy at 99.923%. The authors concluded that XGBoost is the most effective algorithm for detecting credit card fraud.

In a study conducted by (Asha and Suresh Kumar 2021), the authors proposed a novel process for credit card fraud detection using multiple algorithms, including SVM, Artificial Neural Network (ANN), and K-Nearest Neighbor (KNN). The authors conducted experiments using the proposed system with a dataset of 31 attributes related to name, age, account information, and transaction outcomes. They compared the performance of all three algorithms used in the experiment and found that ANN predicts well compared to systems developed using SVM and KNN algorithms (Asha and Suresh Kumar, 2021).

In an enhanced credit card fraud detection model using machine learning, 66 models were tested using a real-world European credit card dataset and stratified K-fold cross-validation. Initially, nine algorithms DT, LR, RF, KNN, SVM, NB, XGBoost, and ANN—were evaluated for detecting fraudulent transactions. The top three performers—Cat-Boost, XGBoost, and RF advanced to the second stage, where nineteen resampling techniques were applied. The All KNN-Cat-Boost model outperformed others, achieving an AUC of 97.94%, a recall of 95.91%, and an F1-Score of 87.40% (Alfaiz N and Fati S, 2022).

Another study conducted by (Sohony et al. 2018) proposed an ensemble learning approach for credit card fraud detection, which combines the strengths of RF and NN algorithms. The study's main findings are that the proposed ensemble method outperforms individual algorithms and other state-of-the-art methods in accuracy, precision, recall, and F1 score. The study also highlights the challenges of handling class imbalances in credit card fraud detection and proposes sampling as a possible solution (Sohony et al., 2018).

(Bagga et al. 2020) aimed to improve the accuracy of credit card fraud detection using data mining techniques. The authors used a dataset of credit card transactions and compared the performance of various classifiers, including LR, NB, KNN, and ensemble learning. They found that ensemble learning and pipelining had the highest accuracy in detecting fraudulent transactions, with a recall rate of 87% and 86%, respectively (Bagga et al., 2020).

These studies collectively indicate a transition from purely algorithmic improvements toward more integrated approaches that combine model design, data balancing, and feature engineering. This perspective aligns with the approach adopted in this study, where multiple techniques are integrated within a unified framework to enhance fraud detection performance and generalization capability.

### 2.2 Imbalanced dataset challenge using machine learning algorithms for credit card fraud detection

The imbalanced dataset of credit card transactions is the most common challenge while implementing machine learning algorithms. This problem occurs when one class has a larger number of instances than the other class. In the case of credit card transactions, the number of legitimate transactions is significantly higher than the number of fraudulent transactions, which affects the performance and accuracy of the implemented machine-learning algorithms.

The importance of addressing class imbalance has been further emphasized in recent research. (Hussain et al. 2024) demonstrated that combining over-sampling and under-sampling techniques can lead to improved model performance, particularly in enhancing recall for minority fraud cases. This supports the adoption of resampling strategies such as RUS, ROS, and SMOTE in fraud detection frameworks.

The study focuses on three widely adopted resampling techniques—Random Under-Sampling (RUS), Random Over-Sampling (ROS), and SMOTE—due to their proven effectiveness, simplicity, and computational efficiency in handling imbalanced datasets. These techniques represent the three main categories of resampling: reduction, duplication, and synthetic generation.

1. **Random Under-Sampling (RUS)**
  Under-sampling involves reducing the number of records in the majority class (Al-Shabi, 2019). To accomplish a balanced training set where the majority and minority classes are equally represented, the method aims to decrease the number of instances in the majority class. This technique, widely adopted for preserving the minority class, involves randomly selecting cases from the majority class to construct the training set (Mrozek et al., 2020).
2. **Random Oversampling (ROS)**
  Oversampling is a sampling method that equalizes the dataset by duplicating samples from the minority class. This technique generates a larger dataset without discarding the initial data. However, in instances of extensive data, this approach can potentially cause overfitting issues and elevate the computational complexity (Singh et al., 2022).
3. **Synthetic Minority Oversampling Technique (SMOTE)**
  SMOTE is considered a state-of-the-art oversampling approach (Shamsudin et al., 2020) and is a data sampling technique that amplifies the representation of the minority class by creating artificial instances around existing ones (Al-Shabi, 2019). This method uses synthetic data points rather than straightforward replications of the minority class (Singh et al., 2022).

## 3 Research model and conceptual framework

**Figure 1:**
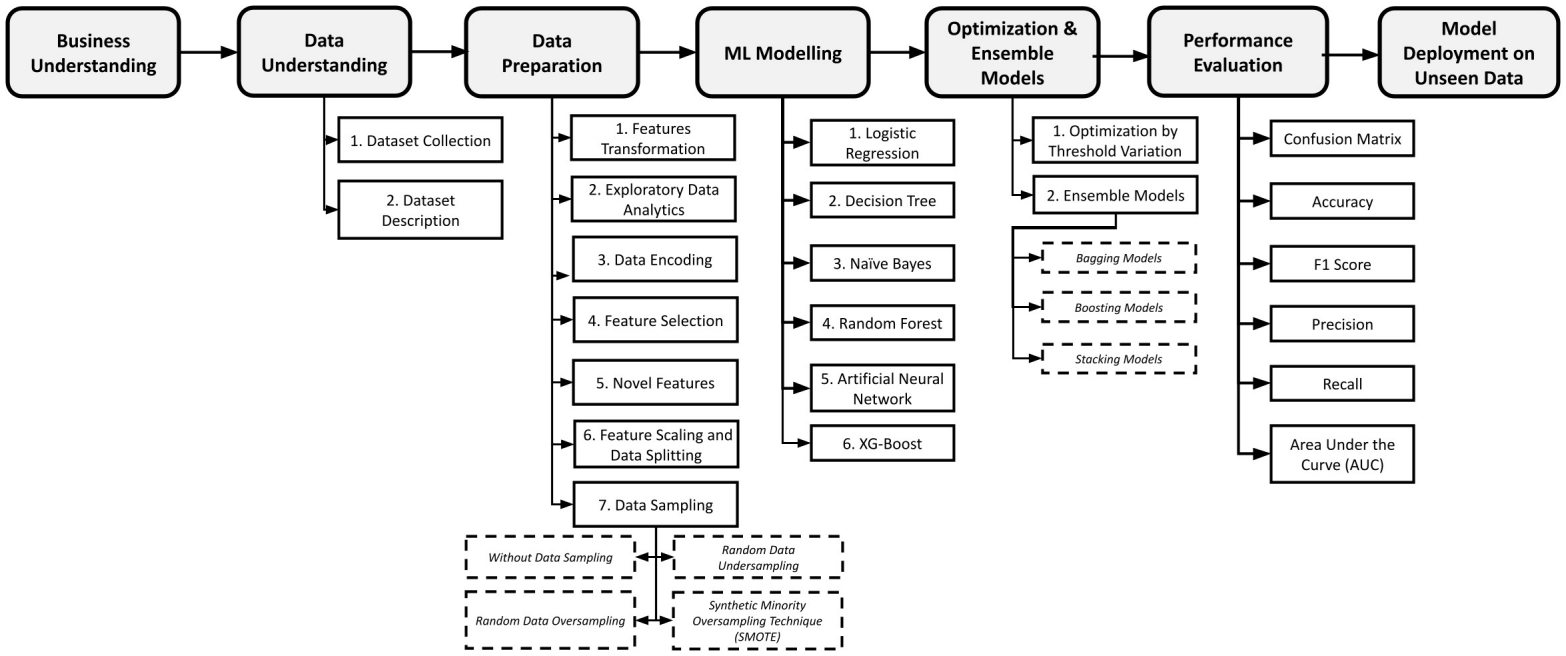
Research conceptual framework.

The Cross Industry Standard Process (CRISP) for Data Mining model was utilized as the research model. The CRISP-DM data mining project lifecycle is segmented into six phases. While the sequence of phases is flexible, the arrows denote primary and frequent dependencies between them (CRISP–DM, 2000). An overview of the conceptual framework is illustrated in Figure 1.

### 3.1 Dataset understanding

1. **Dataset collection**
  The datasets used in this study include both synthetic and real-world data sources. Synthetic datasets (Datasets 1 and 2) provide controlled environments with balanced feature distributions, enabling robust model training. In contrast, real-world datasets (Datasets 3–6) exhibit higher variability, noise, and complex fraud patterns. These differences may affect model performance, as models trained on synthetic data may not fully capture real-world transaction behaviors. Therefore, evaluating models on multiple unseen datasets ensures a more realistic assessment of generalization capability.
  A summary of the datasets is provided below:
  - **Dataset 1**: Credit card fraud detection dataset used as the primary train–test dataset, sourced from Sparkov Data Generation by Brandon Harris (GitHub). It contains 1,300,000 datapoints with 12 categorical and 11 numerical features, and no missing values (Harris, 2020).
  - **Dataset 2**: Credit card fraud detection dataset used as unseen dataset 1, also sourced from Sparkov Data Generation by Brandon Harris (GitHub). It contains 555,719 datapoints with 12 categorical and 11 numerical features, and no missing values (Harris, 2020).
  - **Dataset 3**: Credit card fraud detection dataset used as unseen dataset 2, sourced from a European bank (Kaggle). It includes 284,807 datapoints with 31 numerical features and no categorical features or missing values (Dal Pozzolo et al., 2015).
  - **Dataset 4**: Credit card fraud detection dataset used as unseen dataset 3, sourced from Dhanush Narayanan (Kaggle). It contains 1,000,000 datapoints with 8 numerical features, no categorical features, and no missing values (Narayanan, 2021).
  - **Dataset 5**: Payment fraud detection dataset used as unseen dataset 4, sourced from the PaySim Company (Data World). It includes 10,126 datapoints with 6 categorical and 11 numerical features, and contains some missing values (Lopez-Rojas et al., 2016).
  - **Dataset 6**: Bank fraud detection dataset used as unseen dataset 5, sourced from Volodymyr Gavrysh (Kaggle). It consists of 20,467 datapoints with 114 numerical features, no categorical features, and some missing values (Gavrysh, 2022).
2. **Dataset description**
  Datasets 1 and 2 are synthetic credit card transaction datasets generated using the Sparkov Data Generation tool developed by Brandon Harris. The simulator utilizes a predefined list of customers, merchants, and transaction categories created with the faker Python library. Dataset 1 comprises 1.3 million synthetic transactions originating from 1,000 customers between 1 January 2019 and 31 December 2020, involving 800 merchants. Dataset 2 contains 555,000 transactions and serves as unseen data to test the performance of the model. Fraudulent transactions make up 0.5% of both datasets. Each data set comprises 22 features, both categorical and numerical.

### 3.2 Data preparation

Most datasets are massive and complicated, containing redundant, noisy, and irrelevant information. Thus, data preprocessing is very critical for a successful machine-learning system. Data preprocessing can be achieved by outlier and noise detection, handling missing values, data scaling and normalization, feature selection, and imbalanced data treatment (Alexandropoulos et al., 2019).

**Figure 2:**
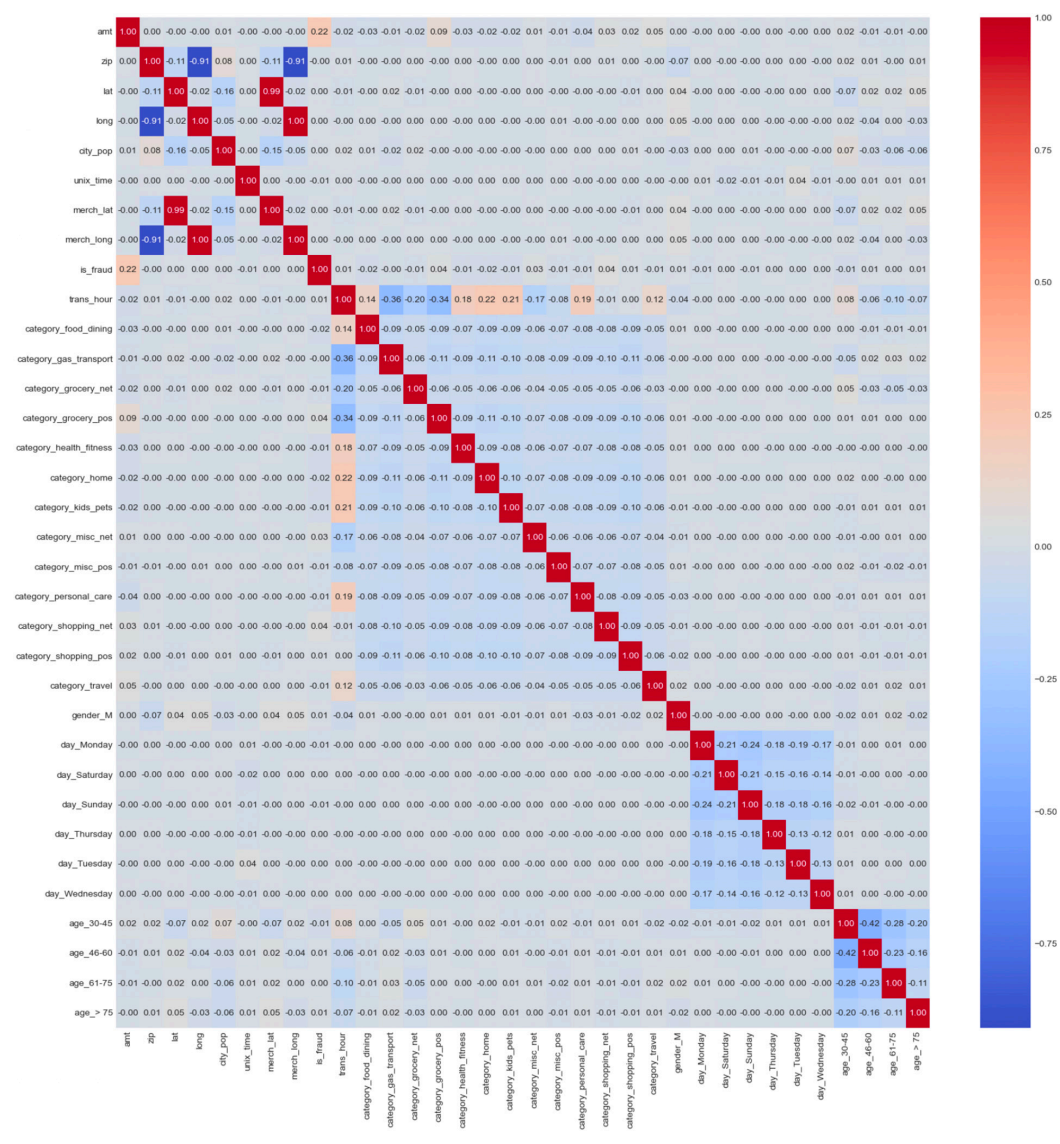
Heatmap of credit card dataset features.

1. **Feature transformation**
  Feature transformation involves the creation of new features derived from existing ones, typically accomplished through mathematical mappings (Dong and Liu, 2018). Several new columns are derived from the transaction timestamp, such as the transaction hour, day of the week, and year-month period. This transformation aims to reduce the number of unique values to enhance the feature correlation.
2. **Data encoding**
  Most machine-learning algorithms cannot process plain text and require numerical input for solving regression and categorical problems. Therefore, encoding the categorical features into a numerical format is necessary. One-hot encoding is a well-known categorical data encoding technique that improves the memory and computing capabilities of machine learning models.
3. **Feature selection**
  Feature selection is selecting the relevant features from the original dataset. The following feature selection techniques are compared for the final feature selection:
4. **Feature selection**
  Feature selection involves identifying the most relevant features from the original dataset to improve model performance and reduce computational complexity. In this study, various filter and wrapper-based techniques were applied, as detailed below:
  - **Filter methods**
    **— Correlation-based feature selection using the heat map scoring**:
      Features with high correlation probabilities are deemed valuable for analysis as they may provide meaningful insights into the dataset. A heatmap in machine learning is often used to visualize a dataset's correlation matrix of features. It provides a graphical representation of how strongly different features are correlated with each other. The heat map for the features is shown in Figure 2.
    **— Univariate selection techniques**:
      * **Variance threshold**: Threshold = 0.1, 0.3, 0.5
      * **ANOVA** : Number of features to select, including encoded entries = 20, 15, 10
      * **Gini index**: Using DT& RF classifications.
  - **Wrapper methods**
    **— Recursive Feature Elimination (RFE)**: Number of features to select, including encoded entries = 20, 15, 10, 6)
    **— Forward Feature Selection (FFS)**: The feature is initially selected based on> 50% occurrence for the selected techniques.
  The feature selection process combines both filter and wrapper methods to balance computational efficiency and predictive performance. Filter methods, such as correlation analysis and variance thresholding, are used to eliminate redundant and low-variance features. Wrapper methods, including Recursive Feature Elimination (RFE) and Forward Feature Selection (FFS), further refine the feature set by evaluating model performance iteratively. This hybrid approach ensures that the selected features contribute meaningfully to model accuracy while minimizing overfitting.
5. **Novel features for customer behavior**
  New novel features are derived from the existing features in the datasets to uncover meaningful patterns and behaviors of each credit card user. These features offer a deeper understanding of the intricate dynamics of credit card transactions. The novel features derived are transaction frequency and transaction time: This feature is generated by first quantifying the 10th and 90th percentiles of transaction hours per user each day. The feature holds a value of 1 for transactions occurring outside the 10th to 90th percentile range and 0 for those within it, enriching the dataset with features related to user behavior and the timing of transactions and anomaly score: This feature contains the anomaly scores using the iForest Features.
  The proposed behavioral features aim to capture deviations from normal transaction patterns at the individual user level. For instance, transaction frequency reflects unusual activity bursts, while time-based features identify transactions occurring outside typical behavioral windows. These features enhance the model's ability to detect subtle anomalies that may not be captured by traditional transactional attributes alone. Experimental results indicate that incorporating these features improves recall and overall detection capability.
6. **Scaling and data splitting**
  In this study, the standard scaler technique was implemented. Standard scaler applies the z-score normalization, transforming each feature to a mean of 0 and a standard deviation of 1 (Raju et al., 2020). Another data preprocessing step for machine-learning models is data splitting. Data is typically divided into two segments, with training conducted on one part and predictive performance tested on the other. Cross-validation is the frequently employed technique to assess the model's predictive performance. In K-fold cross-validation, the data is evenly divided into k segments or folds. Training and testing occur over k iterations, where in each iteration, one fold is reserved for testing, and the model is trained on the remaining folds (Yadav and Shukla, 2016). The data splitting by K-fold was implemented in the train-test data set with four folds. This ensures that 75% of the data is used to train the model.
7. **Data resampling**
  In this study, the model performance was measured without data resampling, data random undersampling, data random oversampling, and Synthetic Minority Oversampling Technique (SMOTE).

### 3.3 Supervised machine learning modeling techniques

The application and comparison of machine-learning models for credit card fraud detection are implemented on standalone models, optimized models, and ensemble models.

### 3.4 Performance evaluation

**Figure 3:**
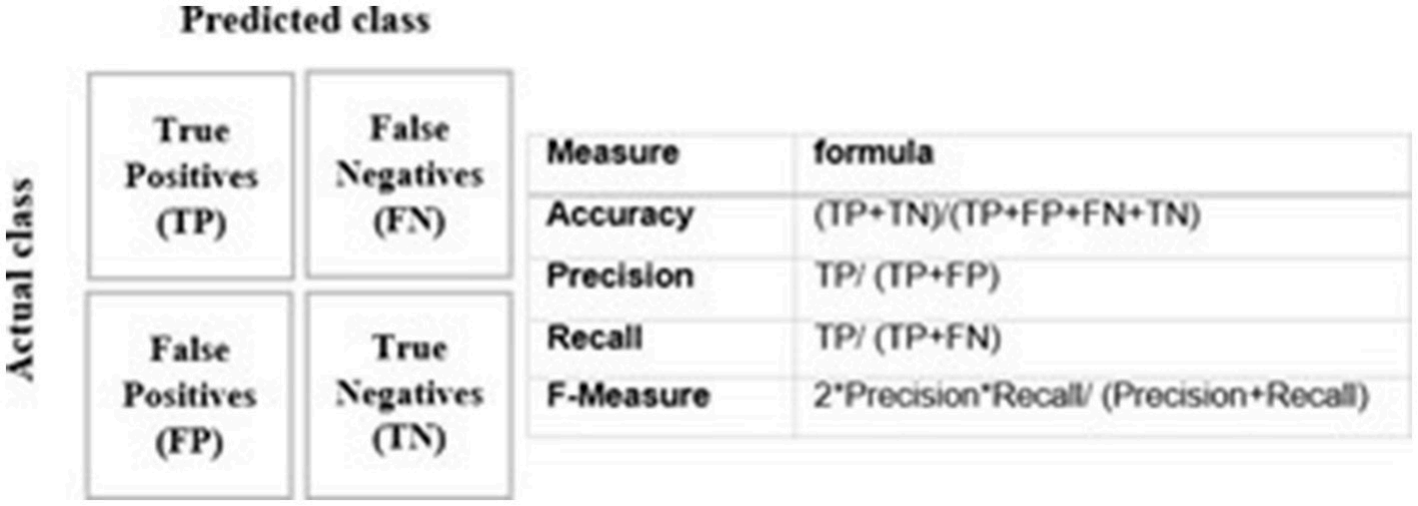
Confusion matrix and performance evaluation for mulas (Dong and Liu, 2018).

Evaluating machine-learning models' performance for credit card fraud detection is critical to ensure their effectiveness in identifying fraudulent transactions. Several common performance evaluation metrics are often discussed in literature reviews, and academic research focuses on credit card fraud detection with imbalanced datasets. These metrics are the Confusion Matrix (CM), Accuracy (A), Precision (P), Recall (R), F1 Score (F1), and Area Under the Curve (AUC) as illustrated in Figure 3.

### 3.5 Best performed model deployment on unseen datasets

After evaluating the results of various machine-learning algorithms, the last step in the research methodology is to select the best algorithm. Then, apply the machine-learning algorithm to other datasets to ensure that the model generalizes well and provides accurate predictions.

## 4 Experimental setup

This section describes the tools used to create the experiment setup of the machine-learning model for credit card fraud detection.

### 4.1 Programming language

A Python object-oriented programming language tool, which is open-source software that offers rich libraries and easy command syntax, is utilized to build the machine learning model for credit card fraud detection. It is widely used for big data and machine-learning projects (Saabith et al., 2019).

### 4.2 Dataset storage

The dataset of credit card transactions is stored in a Microsoft Excel spreadsheet. The data is organized in a tabular structure and saved in a file using the Comma-Separated Values (CSV) file format. CSV is a simple and widely used file format for storing tabular data.

### 4.3 Models parameters selection

Hyperparameters for each model were selected using systematic tuning procedures, including grid search and cross-validation. Consistent tuning strategies were applied across all models to ensure fair comparisons. Parameters such as tree depth, number of estimators, learning rates, and regularization terms were optimized based on validation performance.

## 5 Machine learning models' results

### 5.1 Results of standalone machine learning models

Each model analysis below is performed on the train–test dataset. Four sets of results are obtained for different sampling techniques [No balancing/sampling, Random Under-Sampling (RUS), Random Over-Sampling (ROS), and SMOTE]. The models, such as LR and NB, have weak performance, while others, like DT and NN, have more acceptable performance, and finally, RF and XGBoost have the best performance. No single model can be selected directly, even with the resampling techniques used. Therefore, optimized modeling and ensemble learning techniques are further developed using different configurations. The following methods are used to enhance the model predictions. Results are shown in Tables 1–7.

**Table 1:** Standalone machine learning models performance evaluation.

No.	Model	Acc	F1	Prec	Rec	AUC
0	LR–NB	0.99	0.01	0.08	0.00	0.83
1	LR–RUS	0.87	0.06	0.03	0.76	0.96
2	LR–ROS	0.87	0.06	0.03	0.76	0.91
3	LR–SMOTE	0.87	0.06	0.03	0.76	0.91
4	DT–NB	0.99	0.77	0.76	0.78	0.89
5	DT–RUS	0.96	0.24	0.14	0.97	0.97
6	DT–ROS	0.99	0.78	0.79	0.77	0.88
7	DT–SMOTE	0.99	0.71	0.61	0.83	0.91
8	NB–NC	0.90	0.07	0.04	0.68	0.82
9	NB–RUS	0.63	0.02	0.01	0.77	0.83
10	NB–ROS	0.61	0.02	0.01	0.77	0.82
11	NB–SMOTE	0.61	0.02	0.01	0.77	0.82
12	RF–NB	0.99	0.82	0.88	0.76	0.97
13	RF–RUS	0.97	0.30	0.17	0.97	0.99
14	RF–ROS	0.99	0.80	0.82	0.79	0.97
15	RF–SMOTE	0.99	0.73	0.66	0.82	0.98
16	ANN–NB	0.99	0.83	0.93	0.76	0.99
17	ANN–RUS	0.95	0.20	0.11	0.96	0.99
18	ANN–ROS	0.97	0.35	0.21	0.95	0.99
19	ANN–SMOTE	0.98	0.44	0.29	0.91	0.99
20	XGB–NB	0.99	0.84	0.91	0.79	0.99
21	XGB–RUS	0.97	0.28	0.16	0.98	0.99
22	XGB–ROS	0.98	0.49	0.33	0.96	0.99
23	XGB–SMOTE	0.99	0.67	0.54	0.89	0.99

**Table 2:** Naive bayes model results vs. existing literature.

No.	Model	Acc	F1	Prec	Rec	AUC
This work						
0	NB – No Balancing	0.90	0.07	0.04	0.68	0.82
1	NB – RUS	0.63	0.02	0.01	0.77	0.83
2	NB – ROS	0.61	0.02	0.01	0.77	0.82
3	NB – SMOTE	0.61	0.02	0.01	0.77	0.82
Literature						
0	NB (Alfaiz N and Fati S, 2022) – No balancing	0.99	0.24	0.64	0.14	0.57
1	NB (Bagga et al., 2020)– No balancing	0.99	0.40	0.26	0.85	–

**Table 3:** Logistic regression model results vs. existing literature.

Sr. No.	Model name	A	F1	P	R	AUC
This work						
0	LR – No balancing	0.99	0.01	0.08	0.00	0.83
1	LR – RUS	0.87	0.06	0.03	0.76	0.91
2	LR – ROS	0.87	0.06	0.03	0.76	0.91
3	LR – SMOTE	0.87	0.06	0.03	0.70	0.91
Literature				
0	LR (Alfaiz N and Fati S, 2022) – Without balancing	0.99	0.66	0.64	0.69	0.84
1	LR (Sohony et al., 2018) – No Balancing	0.99	–	0.92	0.64	–
2	LR (Bagga et al., 2020) – No balancing	0.982	0.14	0.07	0.91	–

**Table 4:** Decision tree model results vs. existing literature.

No.	Model	Acc	F1	Prec	Rec	AUC
This work						
0	DT – No Balancing	0.99	0.77	0.76	0.78	0.89
1	DT – RUS	0.96	0.24	0.14	0.97	0.97
2	DT – ROS	0.99	0.78	0.79	0.77	0.88
3	DT – SMOTE	0.99	0.71	0.61	0.83	0.91
Literature						
0	DT (Jain et al., 2020) – No Balancing	0.99	0.79	0.81	0.77	–
1	DT (Alfaiz N and Fati S, 2022) – No balancing	0.99	0.75	0.77	0.74	0.87

**Table 5:** Random forest model results vs. existing literature.

No.	Model	Acc	F1	Prec	Rec	AUC
This work						
0	RF – No Balancing	0.99	0.22	0.88	0.77	0.97
1	RF – RUS	0.97	0.30	0.17	0.97	0.99
2	RF – ROS	0.99	0.80	0.82	0.79	0.97
3	RF – SMOTE	0.99	0.73	0.66	0.82	0.98
4	RF – ROS (threshold = 0.2)	0.99	0.76	0.68	0.86	0.97
Literature						
0	RF (Jain et al., 2020) – No balancing	0.99	0.86	0.97	0.78	–
1	RF (Alfaiz N and Fati S, 2022)– No balancing	0.99	0.85	0.78	0.94	0.97
2	RF (Alfaiz N and Fati S, 2022) – ROS	0.99	0.86	0.78	0.95	0.97
3	RF (Alfaiz N and Fati S, 2022) – SMOTE	0.99	0.86	0.83	0.89	0.95
4	RF (Sohony et al., 2018) – No balancing 1	0.99	–	0.96	0.73	–
5	RF (Sohony et al., 2018)– No balancing 2	0.99	–	0.94	0.74	–
6	RF (Bagga et al., 2020) – No balancing	0.99	0.46	0.31	0.89	–
7	RF (Afriyie et al., 2023) – No Balancing	0.96	0.17	0.09	0.97	–

**Table 6:** Artificial neural network model results vs. existing literature.

No.	Model	Acc	F1	Prec	Rec	AUC
This work						
0	ANN – No Balancing	0.99	0.83	0.93	0.76	0.99
1	ANN – RUS	0.95	0.20	0.11	0.96	0.99
2	ANN – ROS	0.97	0.35	0.21	0.95	0.99
3	ANN – SMOTE	0.98	0.44	0.29	0.91	0.99
Literature						
0	ANN (Asha and Suresh Kumar, 2021) – RUS	0.99	–	0.81	0.76	–
1	FFNN (Sohony et al., 2018) – No balancing 1	0.99	–	0.82	0.82	–
2	FFNN (Sohony et al., 2018) – No balancing 2	0.90	–	0.01	0.96	–
3	FFNN (Sohony et al., 2018) – No balancing 3	0.76	–	0.00	0.97	–
4	ANN (Bagga et al., 2020) – No balancing	0.98	0.15	0.08	0.90	

**Table 7:** XG-Boost model results vs. existing literature.

No.	Model	Acc	F1	Prec	Rec	AUC
This work						
0	ANN – No Balancing	0.99	0.83	0.93	0.76	0.99
1	ANN – RUS	0.95	0.20	0.11	0.96	0.99
2	ANN – ROS	0.97	0.35	0.21	0.95	0.99
3	ANN – SMOTE	0.98	0.44	0.29	0.91	0.99
Literature						
0	ANN (Asha and Suresh Kumar, 2021) – RUS	0.99	–	0.81	0.76	–
1	FFNN (Sohony et al., 2018) – No balancing 1	0.99	–	0.82	0.82	–
2	FFNN (Sohony et al., 2018) – No balancing 2	0.90	–	0.01	0.96	–
3	FFNN (Sohony et al., 2018) – No balancing 3	0.76	–	0.00	0.97	–
4	ANN (Bagga et al., 2020) – No balancing	0.98	0.15	0.08	0.90	–

While high recall is essential in fraud detection, false positives also have significant operational implications. Excessive false alerts may lead to unnecessary transaction blocking and reduced customer satisfaction. Therefore, achieving a balance between recall and precision is critical. The results of this study indicate that the proposed models maintain competitive precision levels while improving recall, making them suitable for real-world deployment scenarios.

### 5.2 Results of models' optimization by threshold variation

Model optimization by threshold variation: The default threshold (0.5) for class assignment may not be optimal for every scenario. Therefore, varying the threshold can enhance the model prediction and optimize specific criteria over others (e.g., increase recall over precision). This approach is tested on RF Models and XGBoost as both are considered ensemble models (RF is bagging, while XGBoost is a boosting technique). Shows in Table 8.

1. **Random forest optimization** The RF model with ROS is optimal for credit card fraud detection. Evaluating performance metrics at various thresholds revealed that while precision and F1 score do not peak at a threshold of 0.2, this configuration achieves a balance between accuracy, precision, and recall.
2. **XGBoost optimization** The XGBoost model with SMOTE is another effective choice for credit card fraud detection. Evaluating performance metrics at a threshold of 0.7 shows a harmonious balance between accuracy, precision, and recall.

**Table 8:** Models optimization by threshold variation performance evaluation.

No.	Model	Acc	F1	Prec	Rec	AUC
0	RF–ROS (threshold = 0.2)	0.99	0.76	0.68	0.86	0.97
1	XGBoost–SMOTE (threshold = 0.7)	0.99	0.70	0.67	0.85	0.99

### 5.3 Results of ensemble models

1. **Ensemble models' description**
  - **Bagging model 1:** Uses RF (No Balancing, RUS, ROS, SMOTE), DT (No Balancing, SMOTE), and ANN (No Balancing, RUS, ROS, SMOTE) with average probability and equal weighting. Employs variations of two model types (DT+RF and ANN) for robustness.
  - **Bagging model 2:** Uses RF (No Balancing, RUS, ROS), NB (SMOTE), LR (SMOTE), DT (No Balancing, SMOTE), and ANN (No Balancing, RUS, SMOTE) with average probability and different weighting. Incorporates diverse models with higher weights for the best-performing models to improve predictions.
  - **Boosting model 1:** Combines DT with Adaptive Boosting (AdaBoost). Integrates weak learners with deep learning for enhanced performance.
  - **Boosting model 2:** Combines LR with AdaBoost.
  - **Boosting model 3:** Combines NB with AdaBoost.
  - **Stacking model 1:** Uses ANN as the meta-model and RF (No Balancing, RUS, ROS, and SMOTE) as the base model. Leverages the strengths of RF (bagging ensemble) and ANN (deep learning model).
  - **Stacking model 2:** Uses RF as the meta-model and XGBoost (No Balancing, RUS, ROS, and SMOTE) as the base model. Combines XGBoost (boosting ensemble) and RF (bagging ensemble) for superior performance.
2. **Ensemble by bagging results** The bagging ensemble models, referred to as *Bagging 1* and *Bagging 2*, demonstrate strong performance in credit card fraud detection. Both models achieved an accuracy above 0.997, indicating high proficiency in class label prediction.
  Bagging 1 slightly outperforms Bagging 2 in terms of F1-score (0.795 vs. 0.791) and precision (0.736 vs. 0.730). However, both models share an identical recall of 0.864, highlighting their equal effectiveness in capturing true positives.
3. **Ensemble by boosting results** The boosting ensemble models, identified as *Boosting 1, Boosting 2*, and *Boosting 3*, show varying performance levels in credit card fraud detection. Boosting 1 (DT with AdaBoost) delivers the highest overall performance, with an accuracy of 0.9977, an F1 score of 0.794, and balanced precision (0.811) and recall (0.778). Its AUC score of 0.927 indicates strong discriminatory power.
4. **Ensemble by stacking results** Stacking ensemble models, defined as *Stacking 1* (RF as base, NN as meta-model) and *Stacking 2* (XGBoost as base, RF as meta-model), also show high performance. *Stacking 1* achieves an accuracy of 0.9979, an F1 score of 0.812, precision of 0.832, and recall of 0.792, indicating a balanced trade-off. Its AUC score of 0.933 underscores its robust overall performance. Stacking 2 has a high accuracy of 0.9971 and an F1 score of 0.777, with a focus on recall (0.864) over precision (0.706). Its AUC score of 0.965 indicates strong discriminatory power. Both models perform exceptionally well, but Stacking 2 excels in the recall, crucial for fraud detection tasks. Results are shown in Table 9.

**Table 9:** Ensemble models performance evaluation.

No.	Model	Acc	F1	Prec	Rec	AUC
0	Bagging–1 (10 models)	0.99	0.79	0.73	0.86	0.99
1	Bagging–2 (10 models)	0.99	0.79	0.72	0.86	0.99
2	Boosting–1 (DT + AdaBoost)	0.99	0.79	0.81	0.77	0.92
3	Boosting–2 (LR + AdaBoost)	0.99	0.00	0.00	0.00	0.82
4	Boosting–3 (NB + AdaBoost)	0.85	0.00	0.00	0.06	0.67
5	Stacking–1 (RF base, NN meta)	0.99	0.80	0.79	0.80	0.93
6	Stacking–2 (XGB base, RF meta)	0.99	0.77	0.70	0.86	0.96

Among the evaluated ensemble approaches, the bagging-based model demonstrated the most consistent performance across multiple evaluation metrics, including accuracy, recall, and AUC. In contrast, boosting and stacking methods showed higher variance and sensitivity to data imbalance in certain configurations. Therefore, the bagging model was selected as the final model for evaluation on unseen datasets due to its superior stability and generalization capability.

### 5.4 Results of deploying best performed model on unseen datasets

Bagging 1 ensemble algorithm is selected as the best-performing algorithm. Bagging 1 was checked against 5 different sets of datasets using novel features for the train–test dataset/unseen dataset 1 (both datasets have the same features). The ensemble model demonstrates acceptable performance across various datasets, showcasing its robustness and effectiveness shown in Table 10.

**Table 10:** Bagging–1 performance evaluation results on unseen datasets.

No.	Dataset	Acc	F1	Prec	Rec	AUC
0	Dataset 1 (train–test)	0.99	0.79	0.73	0.86	0.99
1	Dataset 1 (train–test) + novel features	0.99	0.83	0.77	0.89	0.99
2	Dataset 2 (unseen–1)	0.99	0.61	0.46	0.88	0.99
3	Dataset 2 (unseen–1) + novel features	0.99	0.66	0.53	0.88	0.99
4	Dataset 3 (unseen–2)	1.00	0.84	0.94	0.76	0.98
5	Dataset 4 (unseen–3)	1.00	1.00	1.00	1.00	1.00
6	Dataset 5 (unseen–4)	0.99	0.87	0.93	0.82	0.98
7	Dataset 6 (unseen–5)	0.98	0.96	0.95	0.96	0.99

In comparing NB models developed using various sampling techniques with those documented in existing literature, distinct patterns emerge.

The results show consistency in terms of accuracy with the literature when no balancing technique is used, while this study's results fall short when compared to SMOTE results (Varmedja et al., 2019).

Moreover, the recall is comparable to most literature, with slightly lower performance. For precision, the results are lower in this study compared to most literature but show good matching as well with (Khatri et al., 2020) results.

The difference in performance metrics is expected due to discrepancies in datasets, preprocessing methods, and model configurations across studies, which introduce variability that warrants cautious interpretation. Hence, it can be concluded that this model's performance is comparable to the literature. Refer to Table 11 below for comparison details with the literature.

**Table 11:** Ensemble models performance evaluation on unseen data vs. existing literature.

No.	Model	Acc	F1	Prec	Rec	AUC
0	Bagging–1 (10 models) – Dataset 1	0.99	0.79	0.73	0.86	0.99
1	Bagging–1 (10 models) + Novel Features – Dataset 1	0.99	0.83	0.77	0.89	0.99
2	Bagging–1 (10 models) – Dataset 2	0.99	0.60	0.46	0.88	0.99
3	Bagging–1 (10 models) + Novel Features – Dataset 2	0.99	0.66	0.53	0.88	0.99
4	Bagging–1 (10 models) – Dataset 3	0.99	0.84	0.94	0.76	0.98
5	Bagging–1 (10 models) – Dataset 4	0.99	0.99	0.99	0.99	1.00
6	Bagging–1 (10 models) – Dataset 5	0.99	0.87	0.93	0.82	0.98
7	Bagging–1 (10 models) – Dataset 6	0.98	0.96	0.95	0.96	0.99
Existing literature						
0	Ensemble Model (Sohony et al., 2018) – Bagging	0.99	–	0.85	0.86	–
1	Ensemble Model (Bagga et al., 2020) – Bagging	0.99	0.81	0.76	0.87	–

In comparing RF models developed using various sampling techniques and threshold variations with those documented in existing literature, distinct patterns emerge. The results show consistency in terms of accuracy with the literature. Moreover, the trade-off between precision and recall is comparable to all literature. results show better recall and precision in this study even with RF, ROS, and threshold 0.2. The small difference in recall and precision is expected due to discrepancies in datasets, preprocessing methods, and model configurations across studies, which introduce variability that warrants cautious interpretation. Hence, it can be concluded that this model's performance is comparable to the literature. Refer to Table 8 below for comparison details with the literature (Alfaiz N and Fati S, 2022; Trivedi et al., 2020).

In a comparison of ANN models developed using various sampling techniques with those documented in existing literature, distinct patterns emerge. The results show consistency in terms of accuracy with the literature. Moreover, the recall results are better when compared to most of the literature. For precision, the results are widely spread with a range of 0.11–0.93, with a similar range observed in different literature. The wide difference in precision range is expected due to discrepancies in datasets, preprocessing methods, and model configurations across studies, which introduce variability that warrants cautious interpretation. Hence, it can be concluded that this model's performance is comparable to the literature. Refer to Table 9 below for a detailed comparison with the literature.

Comparing XGBoost models developed using various sampling techniques and threshold variations with those documented in existing literature, distinct patterns emerge. The results show consistency in terms of accuracy with the literature. Moreover, the trade-off between precision and recall is comparable to most of the literature. The results of the research study (Alfaiz N and Fati S, 2022) show slightly better recall and precision in this study, even with XGBoost and SMOTE, with a threshold of 0.7. The small difference in recall and precision is expected due to discrepancies in datasets, preprocessing methods, and model configurations across studies, which introduce variability that warrants cautious interpretation. Hence, it can be concluded that this model's performance is comparable to the literature. Refer to Table 10 below for comparison details with the literature. The Bagging 1 model's performance improves with novel features, as shown across Datasets 1 and 2. For Dataset 1, the model achieves a higher accuracy of 0.99789 with novel features as compared to an accuracy of 0.99742 without selecting novel features, along with a superior F1 score, precision, recall, and AUC. For Dataset 2, the model also outperforms across all metrics, highlighting the benefit of incorporating additional features. Comparing this work to existing literature, the Bagging 1 model demonstrates competitive performance, achieving high accuracy rates (0.980360 to 0.999968) and strong recalls. The AUC scores align with accuracy, highlighting robustness in predicting class probabilities.

Although ensemble models improve predictive performance, they also introduce additional computational complexity. Bagging-based models are relatively scalable due to their parallelizable structure, making them suitable for large-scale datasets. However, real-time deployment may require optimization techniques such as model pruning, distributed computing, or incremental learning to ensure low latency in production environments.

## 6 Recommendation, future work, and conclusion

Based on this study's findings, several recommendations for card fraud detection are proposed. Firstly, advanced feature engineering techniques should be explored to capture patterns indicative of fraud, incorporating sophisticated data preprocessing and user-centric features such as behavior analytics and transaction context. Secondly, further investigation into ensemble learning approaches, especially bagging techniques, is recommended. Practical applications should deploy scalable machine-learning pipelines capable of processing large volumes of transactional data in real time. Continuous model monitoring and adaptation are essential due to the dynamic nature of fraud patterns and evolving adversarial tactics. Collaborative research and data sharing initiatives among researchers, industry stakeholders, and regulatory bodies are crucial for advancing credit card fraud detection and developing more robust and generalizable models while addressing data privacy and security challenges. Addressing the limitations and challenges of implementing machine-learning models for credit card fraud detection opens avenues for future research. Refining resampling techniques to address dataset imbalance is crucial. Future research should focus on fine-tuning model thresholds and decision boundaries to optimize the trade-off between detection accuracy and operational costs, thereby mitigating the impact of false positives on customer experience and organizational resources. It is also important to consider studying the interpretation of machine-learning models. Another area is exploring machine-learning and deep-learning algorithms in detecting the involvement of groups or gangs involved in committing fraud and suspicious activities based on the analysis of complex networks of individuals and transactions. This can be implemented using social network analysis, which was studied in the domain of AML (Shaikh et al., 2021).

This study aimed to apply supervised machine-learning algorithms for credit card fraud detection. CRISP for the data mining model was utilized as the research model. Multiple synthetic and real-world datasets of credit card transactions were used in this work (a total of 6 datasets). Exploratory data analysis, Data sampling, scaling, and encoding were implemented for data pre-processing. Further, several feature selection techniques were used to select features that enter the model, such as filter techniques (correlation-based, Variance threshold, ANOVA & GINI Index) and wrapper methods (recursive feature elimination & forward feature selection). Moreover, novel features were built based on users' behavioral aspects. These features were transaction frequency (number of transactions per hour for each user), transaction time (transactions are within the normal time behavior of the customer or happen at different times), anomaly score for each user, transaction amount, transaction time, and transaction frequency. Various machine-learning models, including LR, DT, NB, NN, RF, and XGBoost, to assess their effectiveness in credit card fraud detection. Ensemble models were then created using the learnings from the above model using different techniques including bagging, boosting, and stacking. The bagging ensemble models demonstrated the best overall performance across the ensemble models, scoring an accuracy of 0.99, a recall of 0.9, and a precision of 0.77. Finally, the selected bagging model for the application was tested on the datasets collected and compared to the literature results. The implementation on different datasets showed good agreement with the literature and was superior in some datasets, scoring accuracy, recall, and precision >0.95. The findings of this research emphasize the effectiveness of supervised machine-learning algorithms for credit card fraud detection and highlight the pivotal role of customized ensemble models.

Despite the promising results, this study has several limitations. First, the use of synthetic datasets may introduce bias and limit the representation of real-world fraud patterns. Second, while multiple datasets were used, further validation on large-scale industrial datasets is required. Third, deploying machine learning models in real-world financial systems presents challenges such as concept drift, regulatory constraints, and integration with existing infrastructure. Future work will focus on addressing these challenges and improving model adaptability.

## Data Availability

The datasets presented in this study can be found in online repositories. The names of the repository/repositories and accession number(s) can be found below: Data will be provided on the request.
